# Integration of ATAC-Seq and RNA-Seq reveals FOSL2 drives human liver progenitor-like cell aging by regulating inflammatory factors

**DOI:** 10.1186/s12864-023-09349-7

**Published:** 2023-05-13

**Authors:** Min Ding, Weijian Huang, Guifen Liu, Bo Zhai, Hexin Yan, Yong Zhang

**Affiliations:** 1 Institute for Regenerative Medicine, Shanghai East Hospital, Shanghai Key Laboratory of Signaling and Disease Research, Frontier Science Center for Stem Cell Research, School of Life Sciences and Technology, Tongji University, Shanghai, 200092 China; 2 Department of Interventional Oncology, Renji Hospital, Shanghai Jiaotong University School of Medicine, Shanghai, 200127 China; 3 Department of Anesthesiology and Critical Care Medicine, Renji Hospital, Shanghai Jiaotong University School of Medicine, Shanghai, 200127 China; 4 State Key Laboratory of Oncogenes and Related Genes, Shanghai Cancer Institute, Renji Hospital, Shanghai Jiao Tong University School of Medicine, Shanghai, China; 5 Shanghai Cancer Institute, Renji Hospital, Shanghai Jiaotong University School of Medicine, Shanghai, 200127 China

**Keywords:** HepLPCs, ATAC-seq, RNA-seq, Aging, Inflammatory response

## Abstract

**Background:**

Human primary hepatocytes (PHCs) are considered to be the best cell source for cell-based therapies for the treatment of end-stage liver disease and acute liver failure. To obtain sufficient and high-quality functional human hepatocytes, we have established a strategy to dedifferentiate human PHCs into expandable hepatocyte-derived liver progenitor-like cells (HepLPCs) through in vitro chemical reprogramming. However, the reduced proliferative capacity of HepLPCs after long-term culture still limits their utility. Therefore, in this study, we attempted to explore the potential mechanism related to the proliferative ability of HepLPCs in vitro culture.

**Results:**

In this study, analysis of assay for transposase accessible chromatin using sequencing (ATAC-seq) and RNA sequencing (RNA-seq) were performed for PHCs, proliferative HepLPCs (pro-HepLPCs) and late-passage HepLPCs (lp-HepLPCs). Genome-wide transcriptional and chromatin accessibility changes during the conversion and long-term culture of HepLPCs were studied. We found that lp-HepLPCs exhibited an aged phenotype characterized by the activation of inflammatory factors. Epigenetic changes were found to be consistent with our gene expression findings, with promoter and distal regions of many inflammatory-related genes showing increased accessibility in the lp-HepLPCs. FOSL2, a member of the AP-1 family, was found to be highly enriched in the distal regions with increased accessibility in lp-HepLPCs. Its depletion attenuated the expression of aging- and senescence-associated secretory phenotype (SASP)-related genes and resulted in a partial improvement of the aging phenotype in lp-HepLPCs.

**Conclusions:**

FOSL2 may drive the aging of HepLPCs by regulating inflammatory factors and its depletion may attenuate this phenotypic shift. This study provides a novel and promising approach for the long-term in vitro culture of HepLPCs.

**Supplementary Information:**

The online version contains supplementary material available at 10.1186/s12864-023-09349-7.

## Background

The liver is the largest and most important metabolic organ and is critical to human health. Around 300 million people in China are affected by liver disease [1–3]. Although liver transplantation can help patients with end-stage liver disease or acute liver failure [4], its applicability is limited by organ shortages and the need for life-long immunosuppressive therapy [5]. Recently, several alternative therapies such as hepatocyte transplantation and artificial and bioartificial liver support systems have been developed [5–7]. Human primary hepatocytes (PHCs) are regarded as the ideal materials for the establishment of bioartificial liver support systems [8] and cell-based therapies [9–11]. However, obtaining sufficient and high-quality functional human PHCs is often impractical due to their limited proliferative capacity in an in vitro environment.

In our previous studies, we established a chemical reprogramming strategy to dedifferentiate mouse and human PHCs into expandable hepatocyte-derived liver progenitor-like cells (HepLPCs) [12, 13]. HepLPCs can be efficiently expanded in vitro, and the expanded cells can be converted back into metabolically functional PHCs [12, 13], providing a reliable cell source for hepatocyte transplantation and cell-based therapies. However, human HepLPCs can only be cultured for 10–15 passages; their proliferative capacity gradually declines, limiting their clinical utility. Functional decline has also been reported in other studies using different dedifferentiation protocols [14–17].

Understanding the mechanisms of functional decline of HepLPCs during passaging is essential to overcome these limitations. Gene transcription could be regulated by controlling the accessibility of transcription factors [18, 19], and several recent studies have reported that chromatin structure can significantly influence hepatocyte proliferation or liver regeneration [20–22]. Considering the important regulatory role of chromatin accessibility during live regeneration, chromatin accessibility profiling may provide a practical approach to identify the key factors involved in the functional decline of HepLPCs. In this study, we integrated gene expression and chromatin accessibility profiles during conversion and long-term culture of HepLPCs and found that FOSL2 regulates inflammatory factors to induce an aging HepLPC phenotype. This work provides a promising solution for the long-term culture of HepLPCs in vitro.

## Results

### Long-term culture of human HepLPCs in vitro displayed cellular aging

**Fig. 1:**
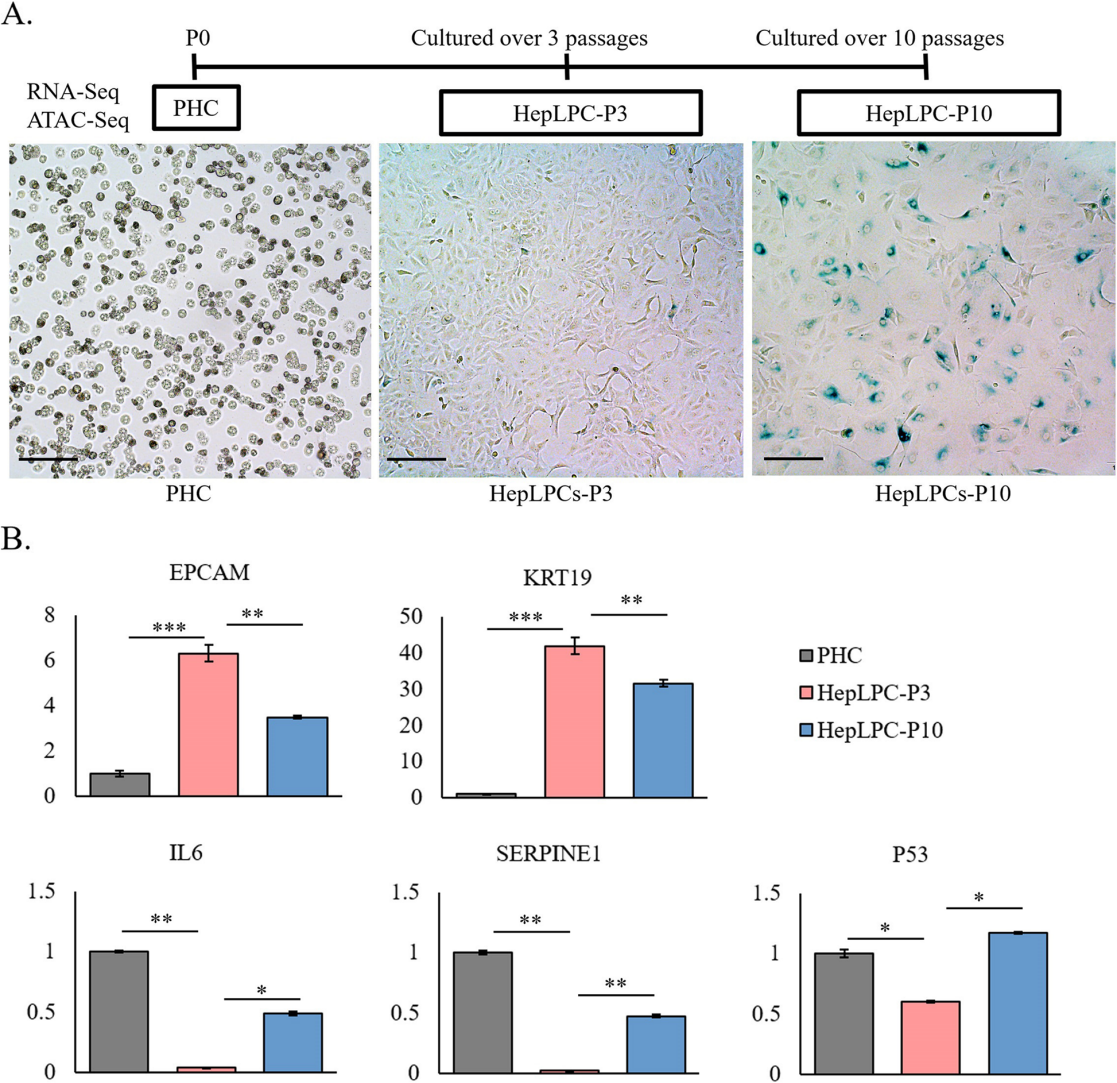
lp-HepLPCs displayed cellular aging. **A** Generation of human hepatocytes (PHCs)-derived liver progenitor-like (HepLPCs) cells in vitro, followed by ATAC-Seq and RNA-Seq. Senescence was detected by senescence β-galactosidase staining at each stage (*N* = 3, and scale bar = 200um). **B** RT-qPCR analyses for the expression of progenitor markers(*EPCAM* and *KRT19*) and aging-related genes (*IL6*, *SERPINE1*, and *P53*). (*p < 0.1, **p < 0.01, ***p < 0.001)

In our previous study, we showed that human PHCs can be converted into HepLPCs under a transition and expansion medium (TEM) culture condition in vitro [12]. However, late-passage HepLPCs (lp-HepLPCs) were found to have reduced proliferative capacity, which may limit the efficient application of HepLPCs. In this study, we found that, under the TEM culture conditions, PHCs were converted into HepLPCs and expanded in vitro; HepLPCs at passage 3 (HepLPC-P3) displayed typical progenitor cell characteristics with a high nucleus/cytoplasm ratio, whereas HepLPCs at passage 10 (HepLPC-P10) showed signs of cellular aging (Fig. 1A). The expression of the progenitor markers *KRT19* and *EPCAM* was significantly increased in HepLPC-P3 and decreased in HepLPC-P10 (Fig. 1B). The expression of the aging-related genes *IL6, SERPINE1,* and *P53* was significantly decreased in HepLPC-P3 and increased in HepLPC-P10 (Fig. 1B). These results demonstrated the cellular aging phenotype of lp-HepLPCs after long-term in vitro culture. To investigate the molecular mechanisms that regulate the proliferative ability of HepLPCs, assay for transposase accessible chromatin using sequencing (ATAC-Seq) and RNA sequencing (RNA-Seq) data from PHC, HepLPC-P3, and HepLPC-P10 were collected and analyzed.

### Transcriptional analysis shows that inflammatory factors are associated with the conversion and aging of HepLPCs

**Fig. 2:**
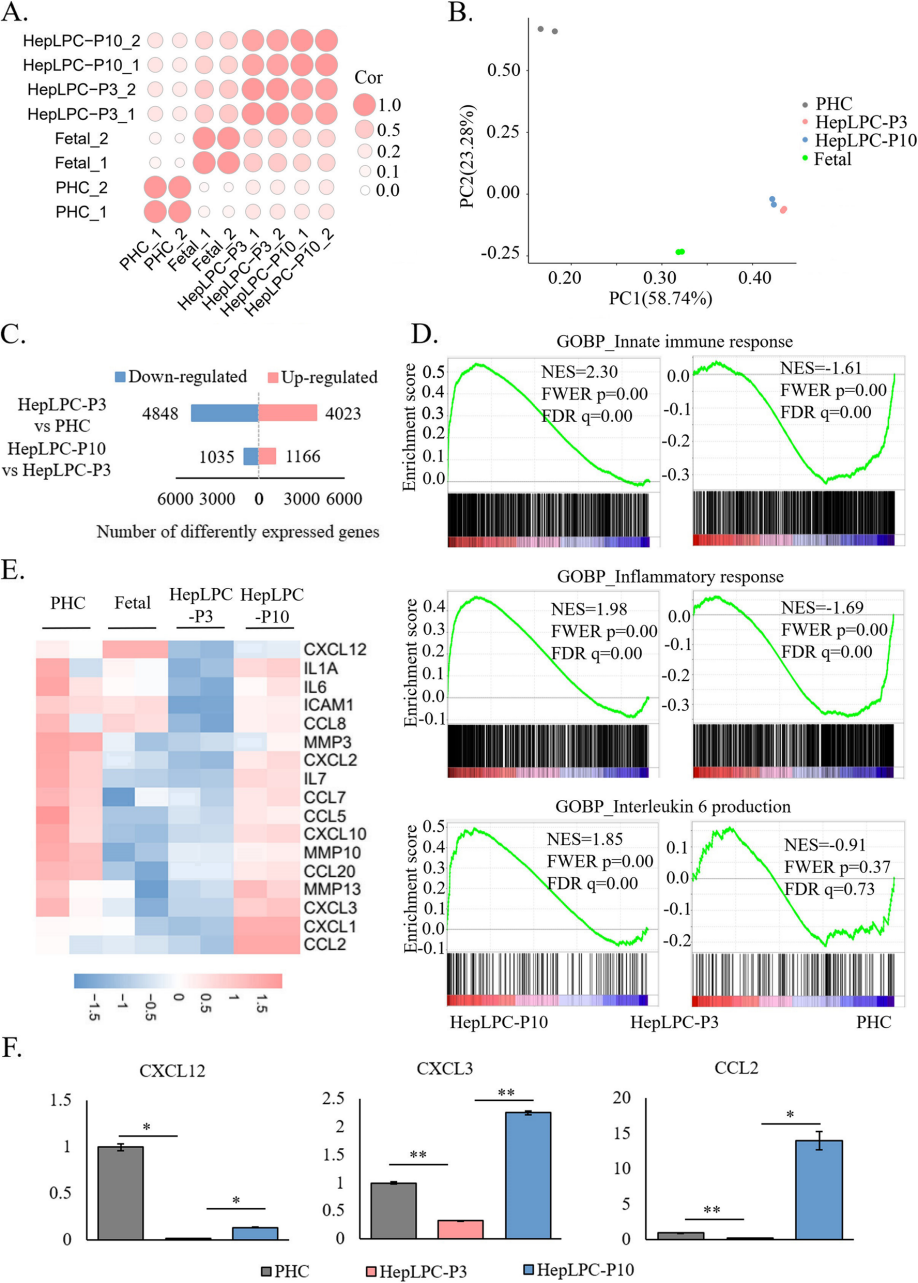
Transcriptional changes indicated the association of inflammatory response with the conversion and aging of HepLPCs. **A** Correlation analysis (Both dot size and dot color represented the level of correlation). **B** PCA analysis. **C** Number of DEGs (padj <  = 0.05 and |log2(FoldChange)|> = 1). **D** Gene set enrichment analysis (GSEA) revealed that processes related to innate immune response, inflammatory response, and interleukin 6 production were repressed in HepLPC-P3 and activated in HepLPC-P10. **E** Heatmap showing the z-scores of log2-transformed expression values of SASP genes based on RNA-seq data. **F** RT-qPCR analysis of chemokine gene expression (*p < 0.1, **p < 0.01, ***p < 0.001)

To conduct genome-wide transcriptional analysis during the conversion and long-term culture of HepLPCs, RNA-Seq data from fetal hepatocytes, PHCs, HepLPC-P3, and HepLPC-P10 were used. Correlations and principal component analyses (PCA) of the whole-genome expression profiles showed that HepLPC-P10 and HepLPC-P3 clustered together, and were close to fetal hepatocytes but separated from PHCs (Fig. 2A and B). Compared to PHCs, HepLPC-P3 showed 4,023 up-regulated genes and 4,848 down-regulated genes. Compared to HepLPC-P3, HepLPC-P10 showed 1,166 up-regulated genes and 1,035 down-regulated genes (Fig. 2C). These comparisons showed that the conversion of PHCs to HepLPC-P3 is associated with large changes in gene expression and relatively fewer changes in gene expression were found between HepLPC-P3 and HepLPC-P10. Using the cell senescence genes from the Human Ageing Genomic Resources (HAGR) [23], GSEA analysis revealed that inhibited cell senescence genes were activated during the conversion of PHCs to HepLPCs, whereas induced cell senescence genes were activated during the aging of HepLPCs, consistent with the changes in cell phenotype (Figure S1A).

Next, the function of differentially expressed genes (DEGs) between different cell states was further investigated. During the conversion of PHCs to HepLPCs, up-and down-regulated genes in HepLPC-P3 significantly overlapped with those in fetal hepatocytes (Figure S1B). Gene Ontology Biology Process (GO-BP) analysis revealed that compared to PHC, the up-regulated genes in HepLPC-P3 and fetal hepatocytes were enriched in processes related to cell proliferation, while the down-regulated genes in HepLPC-P3 and fetal hepatocytes were enriched in processes related to metabolism and other mature hepatocyte functions (Figure S1C). Interestingly, besides processes related to mature hepatocyte functions, inflammatory-related processes, such as the acute-phase response, cytokine-mediated signaling pathway, positive regulation of cytokine production, defense response to virus, and response to xenobiotic stimulus were enriched with the down-regulated genes in HepLPC-P3 and fetal hepatocytes. During HepLPCs aging, DEGs between HepLPC-P10 and HepLPC-P3 significantly overlapped with those between HepLPC-P10 and fetal hepatocytes (Figure S1D). Compared to HepLPC-P3 and fetal hepatocytes, up-regulated genes in HepLPC-P10 were enriched in areas related to inflammatory-related processes, and down-regulated genes in HepLPC-P10 were enriched in areas related to mature liver functions (Figure S1E). These results indicated that HepLPC-P3 exhibited the gene expression pattern of fetal hepatocytes, and the difference in gene expression between HepLPC-P10 and HepLPC-P3 showed a greater deviation from fetal hepatocytes. Notably, during the conversion and aging of HepLPCs, inflammatory response-related genes were significantly down-regulated in HepLPC-P3 and up-regulated in HepLPC-P10 (Figure S1C, S1E). Processes associated with innate immune response, inflammatory response, and interleukin 6 production were inhibited in HepLPC-P3 and activated in HepLPC-P10 (Fig. 2D). Among them, senescence-associated secretory phenotype (SASP) factors were significantly down-regulated in HepLPC-P3 and up-regulated in HepLPC-P10, including chemokines (*CXCL2*, *CXCL3*, *CCL2*, etc.), interleukins (*IL1A*, *IL6*, and *IL7*), proteases and regulators (*MMP3*, *MMP10*, and *MMP13*), etc. (Fig. 2E). The expression of some chemokines was further validated by RT-qPCR (Fig. 2F). Since the expression of chemokine and proinflammatory factor genes was associated with the conversion and aging of HepLPCs, the high expression of these genes in HepLPC-P10 was considered to be the cause of the aging phenotype of lp-HepLPCs.

### Changes in chromatin accessibility correspond to regulation of gene expression

Dynamic changes in chromatin structure and the epigenetic code can affect gene expression and ultimately determine cell state [24]. To identify the key factors regulating the inflammatory response leading to the aging phenotype of lp-HepLPCs, ATAC-seq data was used to correlate the genomic chromatin accessibility and gene expression. All ATAC-seq libraries yielded the expected distribution of fragment lengths, with a majority of nucleosome-free fragments and progressively fewer mono-nucleosomal fragments, indicating good data quality (Figure S2A). The result of the mapped read distributions across the gene bodies also validated the quality of the ATAC-seq (Figure S2B). To identify differentially accessible (DA) chromatin regions, nucleosome-free fragments smaller than 150 bp were used for peak calling. PCA and Pearson correlation analyses were performed on the signals of merged peaks from all samples, showing that the samples were clustered into groups; dynamic changes in chromatin accessibility were observed between PHC and others (Figure S2C-D).

**Fig. 3:**
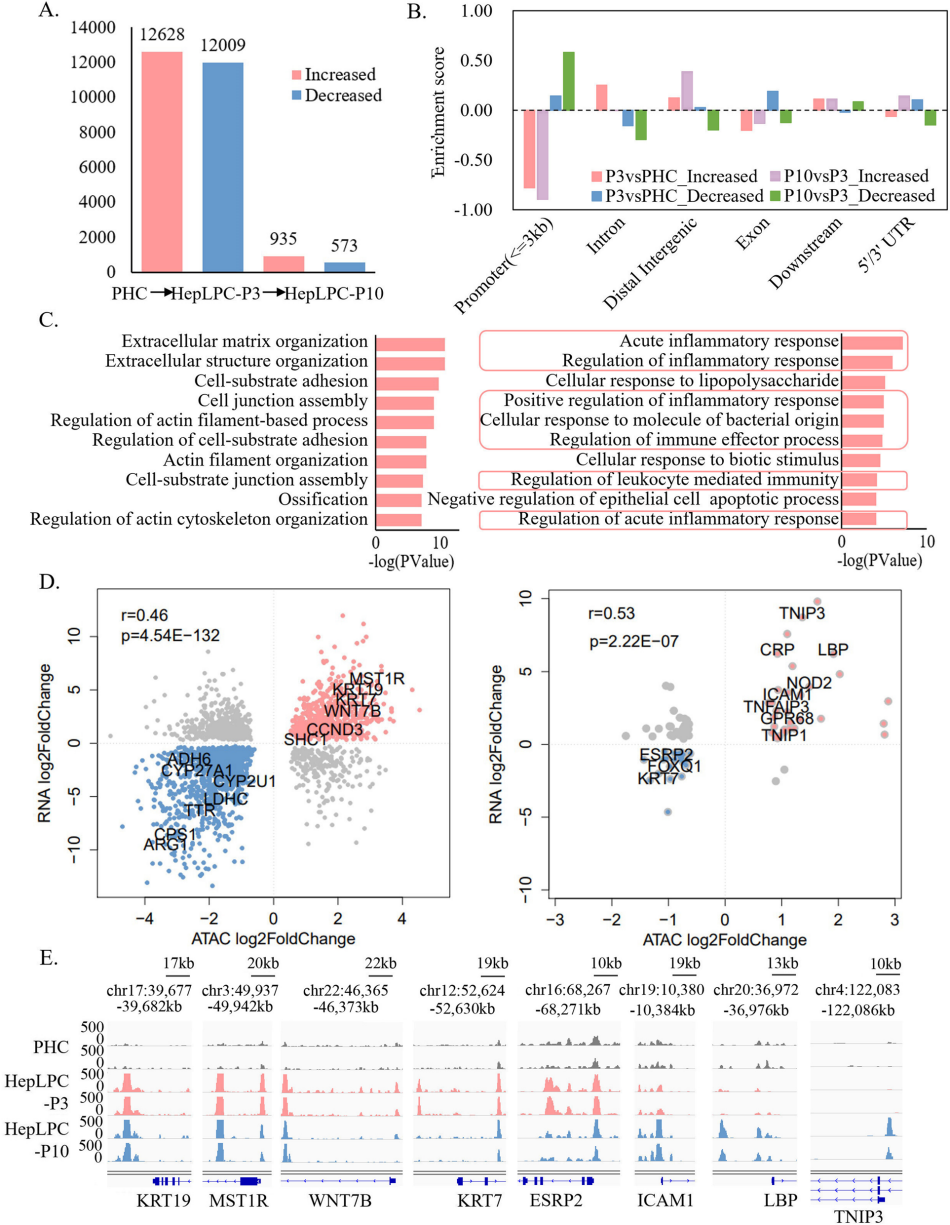
Changes in chromatin accessibility during in vitro culture and association between expression levels and chromatin accessibility. **A** Number of differentially accessible regions between different states (FDR <  = 0.05). **B** Bar plot for the enrichment of the regions with significantly differential accessibility in promoter (< = 3 kb), intron, distal intergenic, exon, downstream and 5’/3’ UTR regions. The enrichment score represents the log2-transformed observed overlapping peaks / the expected overlapping peaks. **C** GO-BP enrichment analysis of increased promoter accessibility in HepLPC-P3 versus PHC (left) and HepLPC-P10 versus HepLPC-P3 (right) indicated that processes involved in cell growth and proliferation were enriched in HepLPC-P3 and processes involved in inflammatory response were enriched in HepLPC-P10. **D** Correlation of the RNA expression and promoter accessibility changes in HepLPC-P3 versus PHC (left) and HepLPC-P10 versus HepLPC-P3 (right) showed that the transcriptomic changes were positively correlated with the changes in promoter accessibility. **E** IGV shows peaks located in the promoter regions of proliferation-related (*KRT19*, *MST1R*, *WNT7B*, *KRT7* and *ESRP2*) and inflammatory-related (*ICAM1*, *LBP*, and *TNIP3*) genes

DiffBind was used to identify DA chromatin regions. 24,637 DA regions were found between PHC and HepLPC-P3, with 12,628 regions showing increased accessibility and 12,009 regions showing decreased accessibility in HepLPC-P3. However, only 1,508 DA regions were found between HepLPC-P3 and HepLPC-P10, with 935 regions showing increased accessibility and 573 regions showing decreased accessibility in HepLPC-P10 (Fig. 3A). This is consistent with the transcriptomic data, where greater differences were observed between PHC and HepLPC-P3 compared to the differences observed between HepLPC-P3 and HepLPC-P10. DA regions with increased accessibility in HepLPC-P3 and HepLPC-P10 were not enriched in promoter regions (Fig. 3B).

Considering the direct effect of promoter accessibility on the transcriptional activity of genes, we further focused on differentially accessible promoter elements. DA regions within 3 kb upstream and downstream of transcription start sites (TSS) were considered as promoter regions, and GO-BP analysis was performed. As expected, processes involved in cell growth and proliferation were enriched among the genes with increased promoter accessibility in HepLPC-P3, whereas processes involved in inflammatory responses were enriched among the genes with increased promoter accessibility in HepLPC-P10 (Fig. 3C). An analysis of the DEGs and their different promoter accessibility showed that the transcriptomic changes were significantly positively correlated with the changes in promoter accessibility in HepLPC-P3 versus PHC (Pearson correlation: r = 0.46, p = 4.54 × 10^–132^) and HepLPC-P10 versus HepLPC-P3 (Pearson correlation: r = 0.53, p = 2.22 × 10^–7^) (Fig. 3D). In HepLPC-P3, cell proliferation-related genes (such as *KRT19, MST1R, WNT7B*, etc.) were up-regulated and showed increased promoter accessibility, while liver function-related genes (such as *ADH6, CYP27A1, CYP2U1*, etc.) were down-regulated and showed decreased promoter accessibility. In HepLPC-P10, inflammatory response-related genes (such as *ICAM1, LBP, TNIP3*, etc.) were up-regulated and showed increased promoter accessibility, while some cell proliferation-related genes (such as *ESRP2, KRT7, FOXQ1*, etc.) were down-regulated and showed decreased promoter accessibility. Promoter accessibility of some proliferation-related and inflammatory-related markers is shown in Fig. 3E. These results corroborated the gene expression profile studies and extended the findings to chromatin accessibility, in that proliferation processes were activated in proliferative HepLPCs (pro-HepLPCs) and inflammatory response processes were activated in lp-HepLPCs.

We then examined the association between chromatin accessibility and gene transcription. DA peaks were found to be located proximal to DEGs in HepLPC-P3 versus PHC (*P* = 8.80 × 10^–23^) and HepLPC-P10 versus HepLPC-P3 (*P* = 2.04 × 10^–11^) (Figure S3A-B). We then compared the gene expression levels in the DA regions with those in the unchanged regions (Figure S3C-D). The association between changes in chromatin accessibility and gene expression in HepLPC-P3 versus PHC and HepLPC-P10 versus HepLPC-P3 showed that changes in promoter and distal accessibility were associated with both gene activation and inhibition in HepLPC-P3 versus PHC (*p* < 2.20 × 10^–16^), whereas only increased promoter (*p* = 1.02 × 10^–6^) and distal accessibility (*p* = 8.81 × 10^–5^) were significantly correlated with the increased gene expression in HepLPC-P10 versus HepLPC-P3. The lack of a significant association between decreased accessibility and inhibition of target genes in HepLPC-P10 versus HepLPC-P3 might be because the target genes were also activated by factors in the TEM.

### TEAD1/2 control the conversion of PHCs into HepLPCs, but are not related to the aging of HepLPCs

**Fig. 4:**
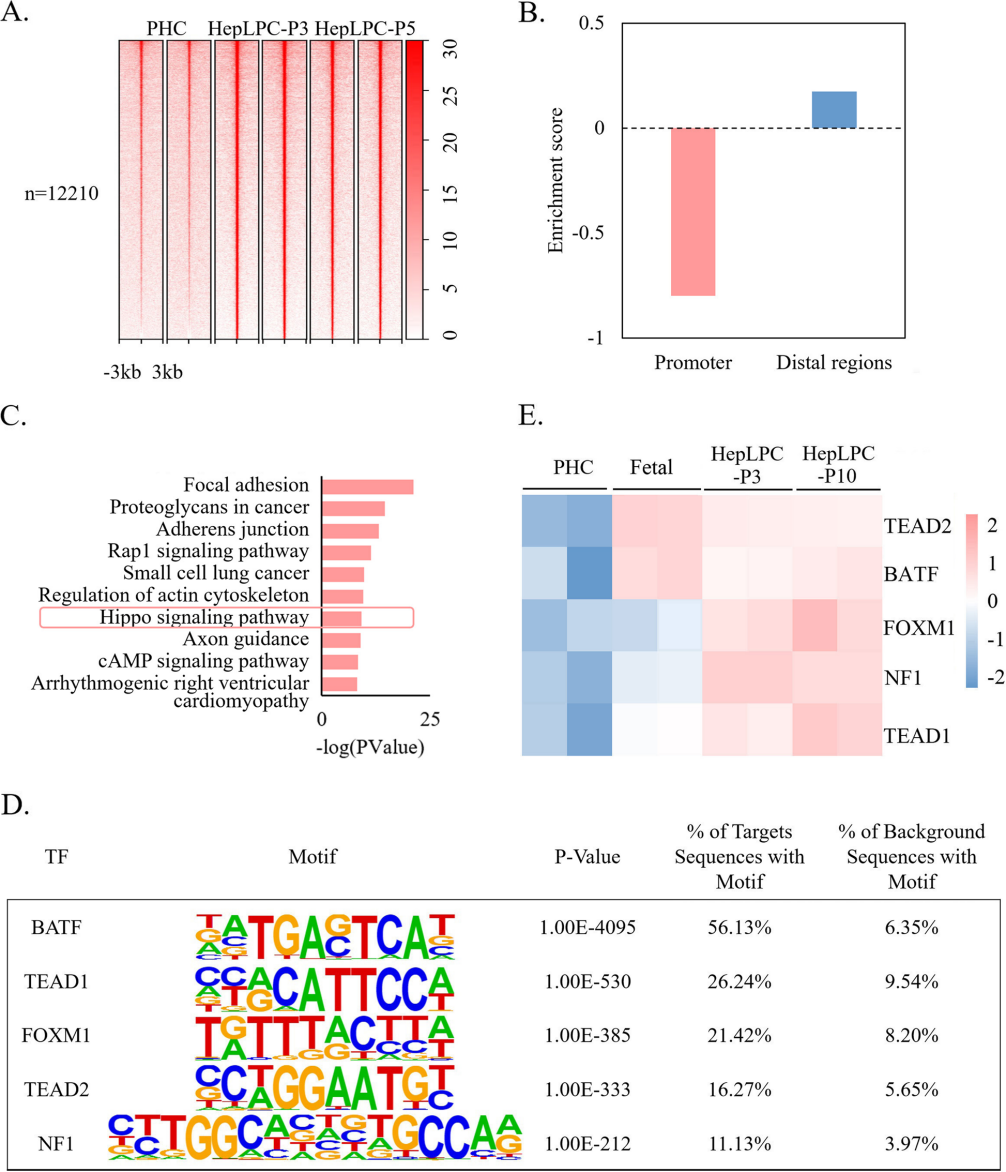
High distal accessibility in HepLPC-P3 and HepLPC-P10 was associated with the conversion of PHCs to HepLPCs. **A** Heatmap of regions with significantly high accessibility in HepLPC-P3 and HepLPC-P10 (FDR <  = 0.05); the heatmap is centered on the ATAC-seq peak (upstream 3 kb and downstream 3 kb of the peaks). **B** Bar plot for the enrichment of the regions with significantly high accessibility in HepLPC-P3 and HepLPC-P10 in promoter (< = 3 kb) and distal regions. The enrichment score represents the log2-transformed observed overlapping peaks / the expected overlapping peaks. **C** KEGG pathway enrichment analysis in genes with high distal accessibility in HepLPC-P3 and HepLPC-P10 indicated that pathways involved in cell growth and proliferation were enriched. **D** Top 5 high-expressed known TFs enriched in HepLPC-P3/P10 high distal accessibility regions. **E** Heatmap showing the z-scores of log2-transformed expression values of the TFs in D based on RNA-seq data

Compared to PHCs, 12,210 ATAC-Seq peaks were identified showing increased accessibility in HepLPC-P3 and HepLPC-P10. And these peaks were preferentially enriched in distal regions, with 10,589 (86.78%) peaks located there (Fig. 4A and B). Given the important regulatory role of enhancers located in distal regulatory regions on gene expression [25], genes with TSS closest to the distal regulatory regions within 100 kb were considered to be their target genes. Functional analysis showed that GO-BPs related to cell migration and cell–cell signaling by wnt were enriched (Figure S4A). In addition, Kyoto Encyclopedia of Genes and Genomes (KEGG) enrichment analysis revealed that pathways involved in cell growth and proliferation were enriched, including the hippo signaling pathway, focal adhesion, and cancer pathways (Fig. 4C). Notably, the hippo signaling pathway has been reported to play an important role in the regulation of stem cell self-renewal and proliferation [26].

To identify DNA binding transcription factors (TFs) that link differential chromatin accessibility and gene expression, motif analysis was performed at distal regions. The top 5 high-expressed known TFs whose motifs enriched in distal regions with increased accessibility in HepLPC-P3/P10 are shown in Fig. 4D, and the normalized expression of the TFs is shown in Fig. 4E. Genes related to cell proliferation, *BATF, FOXM1, NF1, TEAD1, and TEAD2* were found to be enriched and significantly up-regulated in HepLPC-P3 and HepLPC-P10. TEADs have been recognized as key transcription factors of the hippo signaling pathway. Transcriptional coactivators of the hippo signaling pathway, YAP and its paralog TAZ, activate TEAD-mediated transcription and play important roles in organ size control, cell proliferation, and stem cell self-renewal [27–30]. Our results suggest that *TEAD1* and *TEAD2* may play a critical role in the conversion of PHCs to HepLPCs. However, although *TEAD1* and *TEAD2* were highly expressed in HepLPC-P3 and HepLPC-P10, long-term culture led to the aging phenotype; the high expression of *TEAD1* and *TEAD2* in lp-HepLPCs could not maintain cell proliferation. This suggests that other mechanisms are involved in regulating the aging of lp-HepLPCs.

### Inflammatory-related TFs are associated with the aging of lp-HepLPC

**Fig. 5:**
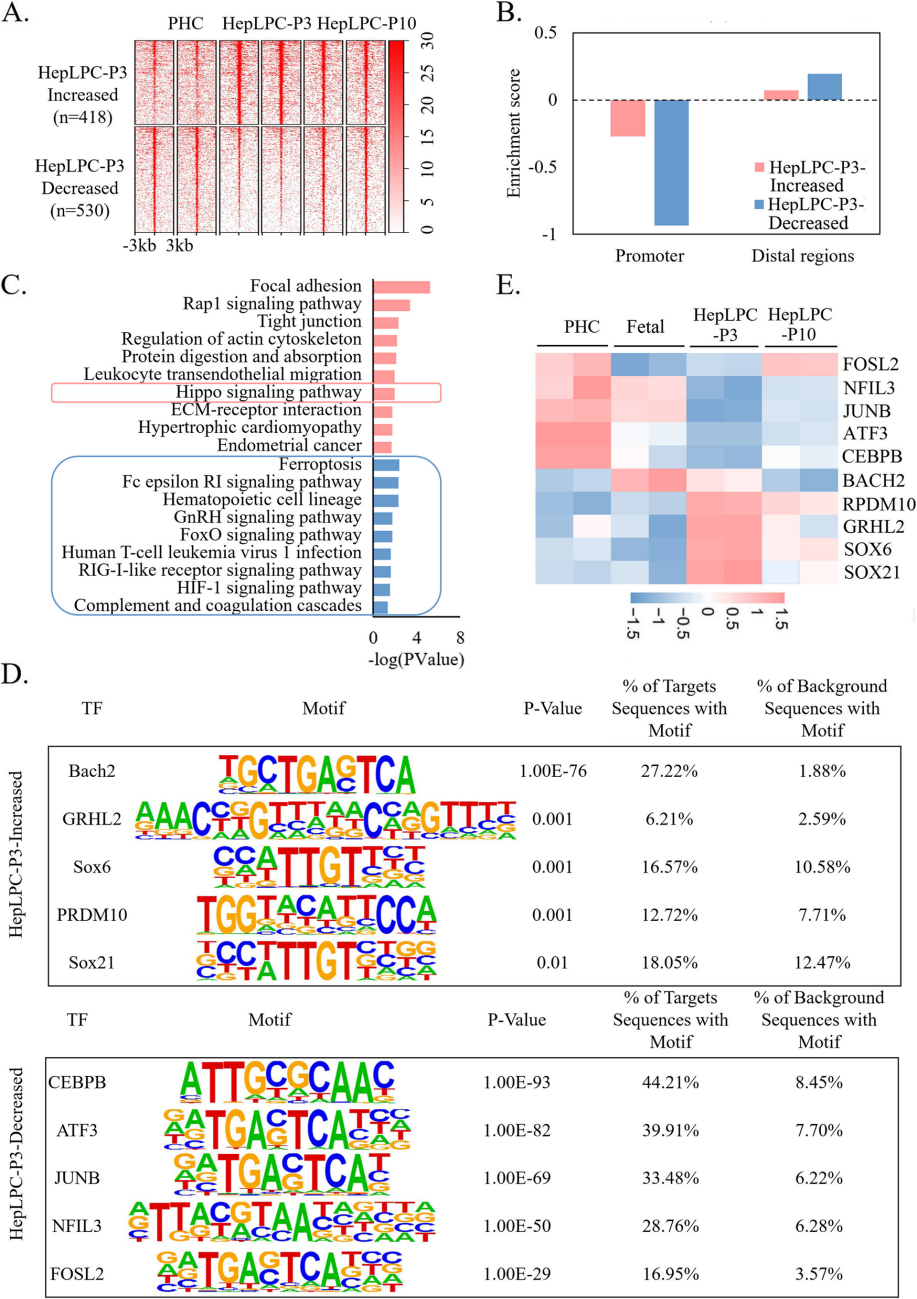
Differential distal accessibility in HepLPC-P3 was associated with the aging of lp-HepLPCs. **A** Heatmap of regions with significantly differential accessibility in HepLPC-P3 comparing both PHC and HepLPC-P10 (FDR <  = 0.05); the heatmap is centered on the ATAC-seq peak (upstream 3 kb and downstream 3 kb of the peaks). **B** Bar plot for the enrichment of the regions with significantly differential accessibility in HepLPC-P3 in promoter (< = 3 kb) and distal regions. The enrichment score represents the log2-transformed observed overlapping peaks / the expected overlapping peaks. **C** Top 10 enriched KEGG pathways of genes with increased (red) or decreased (blue) distal accessibility in HepLPC-P3, indicating that differential distal accessibility in HepLPC-P3 is associated with the aging of lp-HepLPCs. **D** Top 5 up-expressed known TFs enriched in HepLPC-P3-increased distal accessibility regions (top) and down-expressed known TFs enriched in HepLPC-P3-decreased distal accessibility regions (bottom). **E** Heatmap showing the z-scores of log2-transformed expression values of the TFs in D based on RNA-seq data

To investigate the key TFs regulating the aging of lp-HepLPCs, dynamic changes in chromatin accessibility between the different cell types were analyzed. The chromatin accessibility of HepLPC-P3 was compared with that of PHC and HepLPC-P10, respectively. A total of 418 peaks showed higher accessibility in HepLPC-P3 than in PHC and HepLPC-P10, and 530 peaks showed lower accessibility in HepLPC-P3 than in PHC and HepLPC-P10 (Fig. 5A). Both increased and decreased HepLPC-P3 peaks were enriched in distal regions, with percentages over 80% (Fig. 5B). Functional analysis revealed that genes proximal to the increased distal regions in HepLPC-P3 were enriched in processes related to cell migration, whereas genes proximal to the decreased distal regions in HepLPC-P3 were enriched in processes related to cell differentiation and cell growth (Figure S4B-C). Furthermore, KEGG enrichment analysis revealed that the hippo signaling pathway was enriched with genes proximal to increased distal regions in HepLPC-P3, whereas aging-related pathways (Ferroptosis and FOXO signaling pathway) and inflammatory-related pathway (Fc epsilon RI signaling pathway, Hematopoietic cell lineage, RIG-I-like receptor signaling pathway, etc.) were enriched with genes proximal to decreased distal regions in HepLPC-P3 (Fig. 5C).

Motif analysis at distal regions with increased and decreased accessibility in HepLPC-P3 was performed to identify DNA binding TFs associated with the aging of lp-HepLPCs. The top 5 high-expressed TFs whose motifs enriched in distal regions with increased accessibility in HepLPC-P3 and the top 5 down-expressed TFs whose motifs enriched in distal regions with decreased accessibility in HepLPC-P3 are shown in Fig. 5D. The normalized expression of the identified TFs is shown in 5E. Among the regions with increased distal accessibility in HepLPC-P3, *SOX6* and *SOX21* were enriched. *SOX6* and *SOX21* are highly expressed in stem/progenitor cells and are required for stem cell activation and maintenance [31, 32]. Two embryonic-developmental genes, *GRHL2* and *PRDM10*, were found to be enriched. GRHL2 plays an important role in the regeneration of a polarized mucociliary epithelium from basal stem cells [33], and PRDM10 is essential in pre-implantation embryos and embryonic stem cells [34]. However, the most enriched motif, BACH2, has not been reported to be associated with the activation and maintenance of pluripotency; BACH2 has been reported to be associated with immune responses. Deficiency of BACH2 can lead to up-regulation of inflammatory cytokines in CD4 + and CD8 + T cells [35]. In addition, the TFs enriched in the decreased distal regions of HepLPC-P3 were all immune-related genes. CEBPB is an inducible transcription factor that regulates the transcription of SASP genes [36, 37]. NFIL3 is an important transcription factor in the immune system [38, 39]. Other enriched motifs were AP-1 superfamily members (FOSL2 and JUNB) and AP-1-associatied transcription factor (ATF3). AP‑1 activity has been reported to be a major barrier to human somatic cell reprogramming, and AP-1 repressor may improve the efficacy of human reprogramming [40]. Previous work has shown that AP-1 is associated with cell senescence. Depletion of the AP-1 superfamily member cJUN, can suppress the expression of the SASP-like inflammatory response and reactivate the expression of some pro-proliferation genes [41].

Protein–protein interaction network analysis revealed that inflammatory-related TFs, including BACH2, FOSL2, JUNB, ATF3, NFIL3, and CEBPB, were related to each other. Considering the reliable interaction between genes, we identified FOSL2, JUNB, ATF3, and CEBPB as core candidate TFs (Figure S5A). Considering the activation of gene expression and increased promoter and distal accessibility of inflammatory-related genes in HepLPC-P10 compared to HepLPC-P3, the inflammatory response (SASP) was thought to underlie the aging phenotype in lp-HepLPCs. Therefore, the interaction of the core candidate inflammatory-related TFs and SASP factors in Fig. 2E was analyzed using the functional protein association networks STRING [42]. We found that FOSL2, JUNB, ATF3, and CEBPB could regulate the expression of SASP-related genes (Figure S5B).

### FOSL2 depletion delays the aging of lp-HepLPCs

**Fig. 6:**
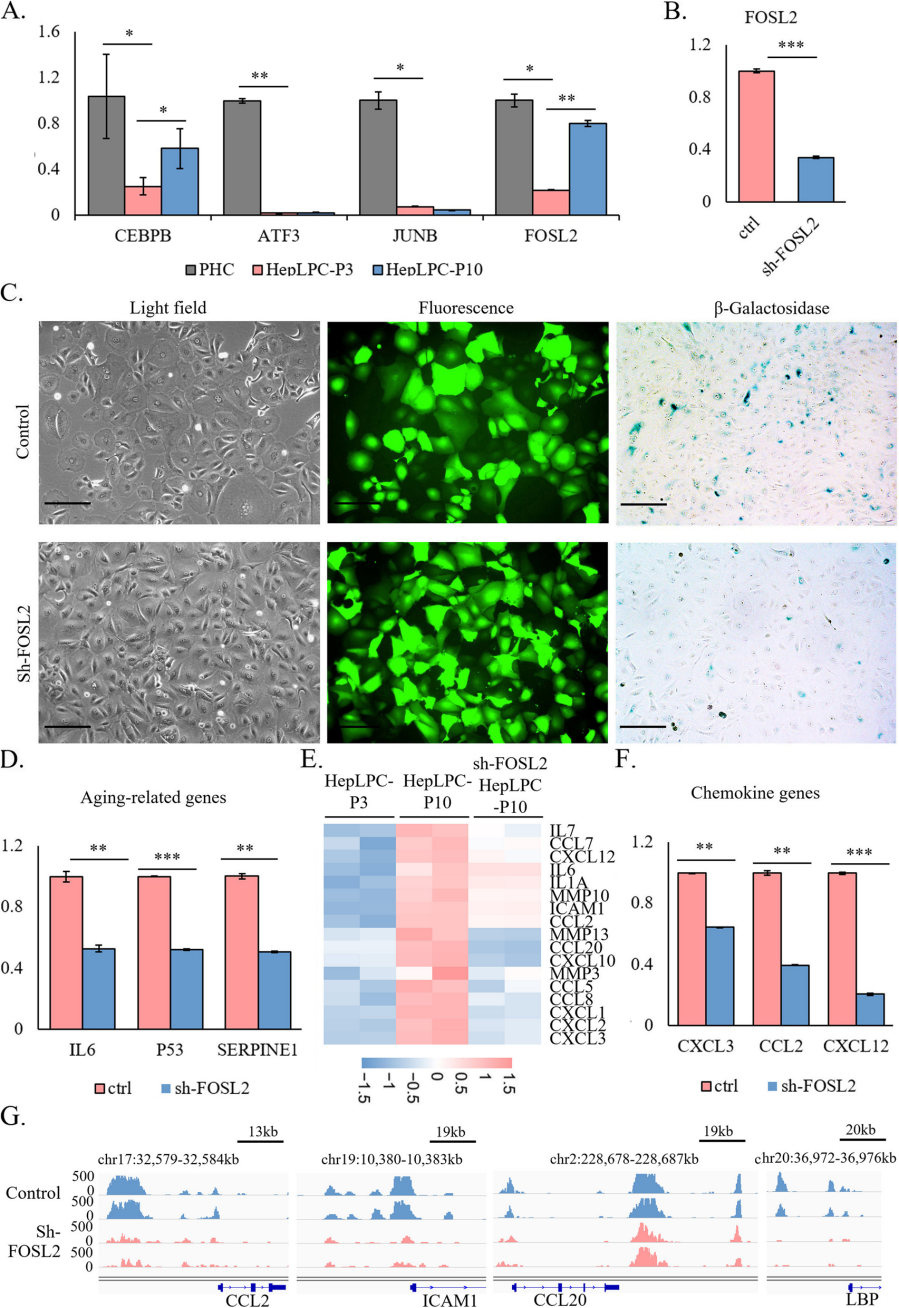
Knockdown of *FOSL2* delayed the aging of HepLPCs. **A** RT-qPCR analysis for the expression of the core TFs during the conversion and aging of HepLPCs. **B** RT-qPCR analysis for the expression of *FOSL2* following shFOSL2 in HepLPC-P3. **C** Light microscopy, fluorescence, and β-Galactosidase staining images of HepLPC-P10 as control versus sh-FOSL2 (*N* = 3, and scale bar = 200um). **D** RT-qPCR analyses for the expression of aging-related genes in FOSL2 silenced HepLPC-P10. **E** Heatmap showing the z-scores of log2-transformed expression values of SASP related genes in sh-FOSL2-HepLPC-P10 compared to HepLPC-P10 and HepLPC-P3 based on RNA-seq data. **F** RT-qPCR analyses for the expression of three chemokine genes in FOSL2 silenced HepLPC-P10. **G** IGV shows peaks located around inflammatory-related (*CCL2*, *ICAM1*, *CCL20* and *LBP*) genes in sh-FOSL2-HepLPC-P10 and HepLPC-P10. (*p < 0.1, **p < 0.01, ***p < 0.001)

To validate the expression changes of the core candidate TFs associated with the aging of HepLPCs, qRT-PCR was used. We found that *CEBPB* and *FOSL2* exhibited similar expression patterns compared to the RNA-seq experiments, however the expression of *JUNB* and *ATF3* did not significantly increase during the aging of HepLPCs (Fig. 6A). Considering the significant changes and high expression level of *FOSL2* in HepLPC-P10 (Figs. 5E and 6A), *FOSL2* was selected to perform interference experiments with short hairpin RNA-mediated *FOSL2* (shFOSL2) knockdown via lentiviral infection to verify its effect on the aging of HepLPCs. HepLPC-P3 were first transfected with the sh-FOSL2 and then compared with sh-GFP as a non-transfected control. After transfection, qRT-PCR showed that the expression of *FOSL2* in HepLPC-P3 was significantly decreased (Fig. 6B). *FOSL2* knock-down (FOSL2-KD) HepLPCs were then passaged to P10. We found that the knowndown of *FOSL2* contributed to a partial attenuation of the aging phenotype in HepLPC-P10 (Fig. 6C). QRT-PCR analysis confirmed that some aging-related genes were significantly down-regulated in *FOSL2*-KD HepLPC-P10 (Fig. 6D). Comparative transcriptomics analyses using RNA-seq data found that compared with HepLPC-P10, up-regulated genes in *FOSL2*-KD HepLPC-P10 were significantly enriched in developmental processes, whereas down-regulated genes in *FOSL2*-KD HepLPC-P10 were significantly enriched in inflammatory-related processes (Figure S6A-B). And GSEA analysis showed that innate immune response, inflammatory response, and interleukin 6 production processes were inhibited in *FOSL2*-KD HepLPC-P10 (Figure S6C). SASP-related genes were significantly reduced to the level of HepLPC-P3 (Fig. 6E), and the downregulated expression of three chemokine-related genes in *FOSL2*-KD HepLPC-P10 was further validated by qRT-PCR (Fig. 6F). Using ATAC-seq data, we found that compared to ctrl HepLPC-P10, 29 (4.14%) regions with significantly differential accessibility were increased accessible regions, whereas 672 (95.86%) were decreased accessible regions in *FOSL2*-KD HepLPC-P10 (Figure S6D). Genes with decreased accessibility in both promoter and distal regions in *FOSL2*-KD HepLPC-P10 were enriched in inflammatory-related processes (Figure S6E). The accessibility of some inflammatory-related genes (such as *CCL2*, *ICAM1*, *CCL20* and *LBP*) is shown in Fig. 6G. In total, these results support that FOSL2 is a key regulator of HepLPCs aging and its depletion can attenuate this phenotypic shift through inflammatory factors.

## Discussion

The in vitro conversion of human PHCs into HepLPCs is an effective method to provide a reliable cell source for cell therapy and drug screening [12]. However, the proliferative potential of human HepLPCs decreases with sequential passaging, limiting their wide clinical and industrial applicability. In this study, we found that long-term culture of HepLPCs in vitro is associated with an aged phenotype. Further study on the molecular mechanism of cell aging during the culture of HepLPCs will provide potential approaches to improve the proliferation of HepLPCs in vitro.

In eukaryotes, gene transcription is a complex regulatory network involving the binding of transcription factors, chromatin structure, epigenetic modifications, and even chromosome interactions [43, 44]. As important regulators of gene expression, transcription factors regulate the expression of target genes by binding to their proximal promoters or distal regulatory enhancers. The binding of regulatory factors to DNA requires the opening of the tight chromatin structure. The study of changes in the open state of chromatin is important for revealing key transcription factors and their regulatory mechanisms. Therefore, in this study, we investigated the relationship between chromatin accessibility and gene expression during the conversion and long-term culture of HepLPCs. By integrating RNA-seq with ATAC-seq, we found that changes in chromatin accessibility were consistent with transcriptome expression. During the conversion of PHCs to HepLPCs, chromatin accessibility and transcriptome expression dramatically change, whereas relatively few changes occur during the aging of HepLPCs in culture (Figs. 2A-C, S2C-D and 3A).We found that the changes in promoter and distal accessibility were associated with both gene activation and inhibition during the conversion of PHCs to HepLPCs, whereas only increased promoter and distal accessibility were found to be significantly correlated with the activated expression of genes during the aging of HepLPCs (Fig. 3D, S3C-D).

During the conversion of PHCs to HepLPCs, cell growth and proliferation-related genes were up-regulated and regulated by increased promoter and distal accessibility. Studies have found that the binding of TFs to DNA occurs mainly in enhancer regions, which are located away from TSS [45]. Therefore, we studied regions of differential distal accessibility and enriched TFs. The hippo signaling pathway, an important pathway regulating stem cell self-renewal and proliferation, was found to be enriched with genes proximal to regions of increased distal accessibility in both HepLPC-P3 and HepLPC-P10. Our findings show that TEAD1 and TEAD2 may be key TF regulators in the conversion of PHCs to HepLPCs. TEAD1 and TEAD2 are likely to regulate the conversion of HepLPCs by targeting enhancers proximal to genes in the hippo/YAP signaling pathway. It is worth noting that bioactive small molecules that regulate the hippo/YAP signaling pathways are included in the TEM culture [12, 13]. However, during long-term culture of HepLPCs, the highly expressed *TEAD1* and *TEAD2* were unable to maintain the proliferation of HepLPCs. During the aging of HepLPCs, genes proximal to regions of increased distal accessibility in HepLPC-P3 were also enriched in the hippo signaling pathway. However, the gene expression was not significantly related to chromatin accessibility (Figure S3D). This could be due to the presence of bioactive small molecules in the TEM that regulate the hippo/YAP signaling pathways. Therefore, the decrease in the chromatin accessibility did not down-regulate the expression of the target genes in HepLPC-P10. These results show that the increased chromatin accessibility in HepLPC-P3 and hippo signaling pathway is not closely related to HepLPC aging.

During HepLPC aging, inflammatory-related processes were found to be activated (Fig. 2D). SASP factors were highly expressed in aged HepLPCs. Activated expression of inflammatory factors may be the cause of HepLPCs aging. Studies have shown that inflammatory factors negatively regulate liver regeneration. The high expression of inflammatory factors in the long-term expanded proliferating human hepatocytes established by Zhang et al. [17] was found to trigger exacerbated macrophage activation which reduced engraftment efficiency [46]. Also, Huang et al. [47] found that CCL5 can negatively regulate liver regeneration; blockade of the chemokine *CCL5* in a partial hepatectomy (PHx) mouse model improved regeneration and greatly optimized survival. In our study, promoter accessibility of inflammatory factors was found to be increased, while distal accessibility of aging- and inflammatory-related genes was also increased in aged HepLPCs. Studying the binding sites of differential distal accessibility regions is expected to identify the key TFs that regulate the inflammatory responses that lead to HepLPCs aging. Motif analysis revealed enrichment of some inflammatory-related TF binding sequences in regions with decreased and increased distal accessibility in HepLPC-P3 (Fig. 5D). CEBPB is an inducible transcription factor that regulates SASP gene transcription [36, 37]. *FOSL2*, *JUNB*, and *ATF3* are members or related genes of the AP-1 superfamily, which has been reported to be a major barrier to human somatic cell reprogramming [40] and to regulate cell aging through the inflammatory response process [41]. In addition, studies have shown that FOSL2 activates T cells [48] and that BACH2 is a master repressor of cytokine genes and is involved in T cell memory [49]. NFIL3 has been reported to be an important transcription factor in the immune system [38, 39]. Our protein–protein interaction analysis revealed that FOSL2, JUNB, ATF3, and CEBPB had a reliable interaction relationship and could regulate SASP. Therefore, FOSL2, JUNB, ATF3, and CEBPB were considered as core candidate TFs that regulate inflammatory factors leading to the aging of HepLPCs.

Further interference experiments confirmed that the effect of *FOSL2*-KD contributed to a partial improvement of HepLPC-P10 aging, with a reduced expression of aging- and SASP-related genes. FOSL2 is a key regulator of HepLPCs aging and its depletion can attenuate this phenotypic shift through inflammatory factors. However, *FOSL2* depletion could only delay but not prevent the aging of HepLPCs.

## Conclusions

In conclusion, integrated analyses of ATAC-seq and RNA-seq data were used to investigate changes in chromatin accessibility and gene expression during the conversion and long-term culture of HepLPCs. Analyses of these two large datasets revealed candidate key TFs that regulate the conversion and aging of HepLPCs. And we found that FOSL2 might drive the aging of HepLPCs by regulating inflammatory factors, and its depletion can attenuate this phenotypic shift. This study provides novel data for understanding the mechanisms of long-term proliferation and aging in in vitro cultured HepLPCs.

## Methods

### Human HepLPCs culture

Liver tissues (1–5 g) was obtained from residual normal liver tissue after transplantation surgery at our hospital. The Ethics Committee of the Hospital approved the use of this material for research purposes, and informed consent was obtained from all participants. Primary human hepatocytes (PHCs) were isolated from these tissues using our previously reported method [12]. Purified hepatocytes were then plated on a tissue culture dish at 0.5–2 × 10^4^ cells per cm^2^ and cultured in TEM [13]. The medium was then changed every other day. Six days after the initial plating, clonal cells were passaged at a ratio of 1:3 after dissociation with Accutase (eBioscience). PHCs, pro-HepLPCs (passage 3, HepLPC-P3), and lp-HepLPCs (passage 10, HepLPC-P10) were collected for RNA-seq and ATAC-seq analysis. For each time point, two technical replicates were prepared for each analysis.

### Cell senescence

SA-β-gal staining was used for cell senescence analysis. Cells were fixed in 4% paraformaldehyde and stained using the senescence β-Galactosidase staining kit (Beyotime, Shanghai, China).

### Quantitative real-time PCR (RT-qPCR)

TRIzol reagent (Absin, Shanghai, China) was used to extract total RNA. Then the quantity and quality of the RNA was determined using a SpectraMax Plus 384 enzyme-labeling instrument. Reverse transcription reactions were performed using Prime Script RT Master Mix (Promega) according to the manufacturer’s instructions. In addition, real-time PCR was performed using a LightCycler® 96 Real-Time PCR System (Roche) and SYBR Green PCR kit (Roche). Gene expression was evaluated using the 2^−ΔΔCt^ method and normalized to the housekeeping gene actin beta (*ACTB)*. The sequences of the utilized PCR primers used are listed in Supplementary Table S1 and were purchased from Sangon Biotech (Shanghai).

### RNA-seq

Total RNA was isolated from the cells using a RNeasy mini kit (Qiagen, Germany). Paired-end libraries were prepared using the TruSeq® RNA Sample Preparation Kit (Illumina, USA) according to the manufacturer’s instructions. Transcriptome sequencing was performed on the Illumina platform (Illumina NovaSeq 6000, Novogene Bioinformatics Technology Co., Ltd., Beijing, China) and 150 bp paired-end reads were generated. Raw sequencing reads were trimmed to remove low-quality reads and adapters using fastp (version 0.22.0). STAR (version 020,201) was used to map the cleaned reads to the hg19 reference genome (GRCh37) with default parameters. Read counts for each gene were summarized using GFOLD (version V1.1.4). DESeq2 was employed to normalize gene expression and detect differentially expressed genes (|log2(FoldChange)|≥ 1, adjusted *p*-values ≤ 0.05). ClusterProfiler (V3.18.1) [50] and Gene set enrichment analysis (GSEA, https://www.gsea-msigdb.org/gsea/index.jsp) [51] were used for functional enrichment analysis of the Gene Ontology Biology Process for DEGs.

### ATAC-seq

ATAC-seq libraries were generated following a previously published protocol [52]. Firstly, nuclei isolated from approximately 10,000 cells were incubated with the Tn5 transposase and tag mentation buffer for 30 min at 37 °C, and then column purified with a MinElute PCR Purification Kit (Qiagen, 28,004). PCR was performed to amplify the ATAC-seq libraries by adding two different barcodes. After the PCR reaction, the libraries were purified with Agencourt Ampure XP beads and sequenced on an Illumina NovaSeq 6000 sequencer using 150 bp paired-end sequencing. Raw sequencing reads were trimmed to remove low-quality reads and adapters using fastp (version 0.22.0). Clean data were aligned to the hg19 reference genome using the Bowtie2 (version 2.4.2) with the options: –local -N 1 -X 2000, followed by removal of PCR duplicates and mitochondrial DNA was carried out. Only concordantly aligned pairs in the pair-end sequence data were used. The Reads Per Kilobase per Million mapped reads (RPKM) normalization method of deepTools bamCoverage [53] was used to transform alignment bam files into read coverage files (bigwig format). The ChIPQC R package was used for quality assessment. Unique reads from fragments that were < 150 base pairs were used for peak calling by MACS2 [54] with the parameters “-p 0.001 –nomodel –shift -75 –extsize 150 –to-large –SPMR”. The Integrative Genomics Viewer (IGV 2.8.2) was used for data visualization. Differential accessible peaks were identified by using the DiffBind R package [55]. Read counts for each region were quantified with dba.count (bUseSummarizeOverlaps = TRUE) and normalized with dba.normalize (normalize = DBA_NORM_TMM), and then differential analysis was performed by using dba.analyze (method = DBA_DESEQ2). Peaks with FDRs of ≤ 0.05 were identified as significant differentially accessible regions. Motif enrichment analysis was performed using the HOMER function findMotifsGenome.pl with default options [56], and motifs with a *p* value less than 0.01 were considered as enriched motifs. The enriched motifs with the expression level of their corresponding TFs and the accessibility of their binding regions changed in the same direction were then identified.

### Integrative analysis of RNA-seq and ATAC-seq data

To evaluate the association between chromatin accessibility and gene expression changes, the ChIPseeker R package [57] was used to annotate the differentially accessible peaks to the nearest TSS. Regions within 3 kb upstream and downstream of the TSS were defined as promoter regions and others were defined as distal regions. The genes with TSS closest to the accessible regions within 100 kb were considered to be their target genes. Pearson correlation (cor) was used to evaluate the relationship between promoter region accessibility and corresponding gene expression at different stages, and a *p*-value ≤ 0.05 was considered to indicate a significant correlation. Next, to investigate the regulation of chromatin accessibility of genes, GO-BP and KEGG pathways were used to perform enrichment analyses using ClusterProfiler (V3.18.1) [50].

### Protein–protein interaction analysis

To investigate the regulatory relationship between transcription factors (TFs) and genes, the functional protein association networks STRING [42] was used to perform the protein–protein interaction analysis. Interaction information from STRING database represent a combination of different data source, including text mining, experiments, databases, co-expression, neighborhood, gene fusion and co-occurrence. The minimum required interaction score was set as medium confidence (0.400) when performing network analysis of TFs and genes. High confidence (0.700) was used to identify core candidate TFs.

### FOSL2 knockdown

Oligo encoding shRNAs specific for human*FOSL2* and scrambled shRNA sequences were ligated into the lentiviral backbone LV-U6-PGK/EGFP/T2A/Puro (OBIO Biosciences, Inc). The packaging plasmids pCMV-VSVG, pMDLg/pRRE, and pRSV-REV were used to transfect the lentivirus into HEK-293 T cells. The supernatant containing the lentiviruses was harvested and filtered through 0.45 μm PVDF filters 48 h after transfection. The virus was concentrated 100-fold by ultracentrifugation (2 h at 60,000 g) and the virus-containing pellet was resuspended in HBSS.

### Statistical analysis

For the comparison of gene expression based on RT-qPCR analyses between two different states, a one-tailed unpaired t-test was used to calculate statistical significance. For the integrative RNA-seq and ATAC-seq analysis, a hypergeometric test was used to determine the significance of overlapping gene sets, and a one-tailed independent t test was used to compare the difference between gene expression foldchanges in differentially accessible promoter and distal regions.

### Data availability

The original data for this study have been submitted to the Genome Sequence Archive (GSA) [58] at the Beijing Institute of Genomics (BIG) Data Center (https://bigd.big.ac.cn/gsa) (accession number: HRA003006). The transcriptomic data of fetal hepatocytes (GSM1707674 and GSM1707675) were included for gene expression profile analyses.

## Supplementary Information

**Additional file 1: Figure S1.** Functional analysis of DEGs between different cell states, related to Figure 2. **Figure S2.** Quality assessment of ATAC-Seq data, related to Figure 3. **Figure S3.** Association of expression levels and chromatin accessibility, related to Figure 3. **Figure S4.** GO-BP analysis of genes with significantly differential distal accessibility among different cell states, related to Figure 4-5. **Figure S5.** Protein-protein interaction networks of TFs. **Figure S6.** Functional analysis of DEGs between sh-FOSL2 affected HepLPC-P10 and ctrl HepLPC-P10, related to Figure 6. **Table S1.** Primer sequences used for RT-qPCR.

## Data Availability

The original data for this study have been submitted to the Genome Sequence Archive (GSA) (58) at the Beijing Institute of Genomics (BIG) Data Center (https://bigd.big.ac.cn/gsa) (accession number: HRA003006). The transcriptomic data of fetal hepatocytes were GSM1707674 and GSM1707675.

## Abbreviations

PHCs: Primary hepatocytes
HepLPCs: Hepatocyte-derived liver progenitor-like cells
ATAC-seq: Assay for transposase accessible chromatin using sequencing
RNA-seq: RNA sequencing
TEM: Transition and expansion medium
pro-HepLPCs: Proliferative HepLPCs
lp-HepLPCs: Late-passage HepLPCs
GSEA: Gene set enrichment analysis
DEGs: Differentially expressed genes
HepLPC-P3: HepLPCs at passage 3
HepLPC-P10: HepLPCs at passage 10
PCA: Principal component analysis
GO-BP: Gene Ontology Biology Process
SASP: Senescence-associated secretory phenotype
DA: Differentially accessible
TSS: Transcription start sites
TFs: Transcription factors
*FOSL2*-KD: *FOSL2* Knock-down
