# Supplementary material for: Integration of ATAC-Seq and RNA-Seq reveals FOSL2 drives human liver progenitor-like cell aging by regulating inflammatory factors
Source: BMC Genomics. 2023 May 13;24:260. doi: 10.1186/s12864-023-09349-7 (PMC10182660; doi:10.1186/s12864-023-09349-7)
Supplement: Supplementary file 1 — Additional file 1: Figure S1. Functional analysis of DEGs between different cell states, related to Figure 2. Figure S2. Quality assessment of ATAC-Seq data, related to Figure 3. Figure S3. Association of expression levels and chromatin accessibility, related to Figure 3. Figure S4. GO-BP analysis of genes with significantly differential distal accessibility among different cell states, related to Figure 4-5. Figure S5. Protein-protein interaction networks of TFs. Figure S6. Functional analysis of DEGs between sh-FOSL2 affected HepLPC-P10 and ctrl HepLPC-P10, related to Figure 6. Table S1. Primer sequences used for RT-qPCR. [file 12864_2023_9349_MOESM1_ESM.docx]

**Supplementary Figures**

**
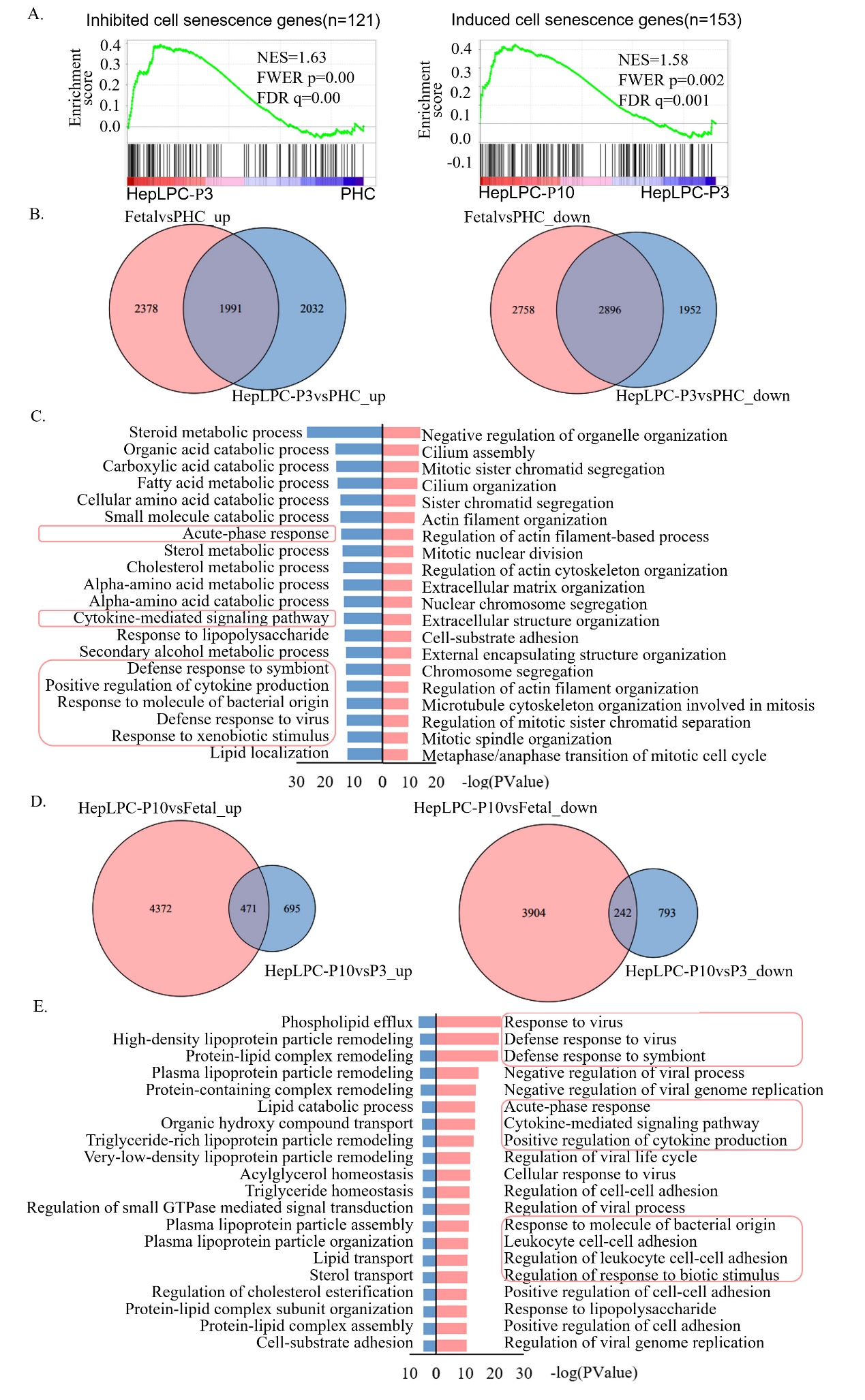
**

**Figure S1** Functional analysis of DEGs between different cell states, related to Figure 2. A. Gene set enrichment analysis (GSEA) of cell senescence genes in human ageing genomic resources (HAGR) during the conversion and aging of HepLPCs. B. Venn diagram showing the common DEGs between HepLPC-P3 and fetal hepatocytes compared to PHC (up-regulated and down-regulated genes; hypergeometric test p-value=0). C. GO-BP enrichment analysis of common up-regulated and down-regulated genes in HepLPC-P3 and fetal hepatocytes compared to PHC (red = up-regulated; blue = down-regulated). D. Venn diagram showing the common DEGs between HepLPC-P10 compared to PHC and fetal hepatocytes (up-regulated genes; hypergeometric test p-value=1.46 x10^-54^. Down-regulated genes; hypergeometric test p-value=3.15 x10^-6^). E. GO-BP enrichment analysis of common up-regulated and down-regulated genes in HepLPC-P10 compared to both fetal hepatocytes and HepLPC-P3 (red = up-regulated; blue = down-regulated).

**
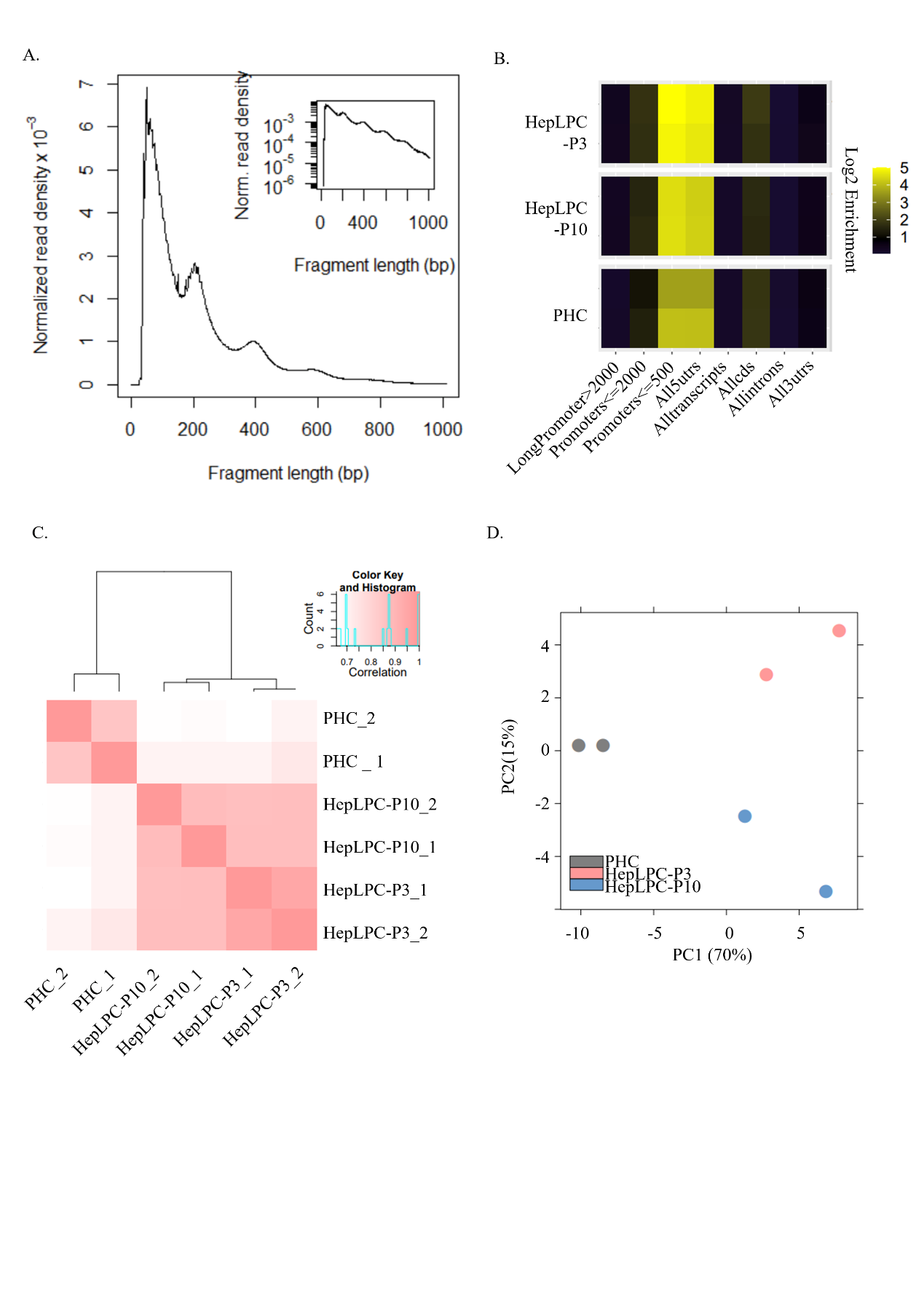
**

**Figure S2**. Quality assessment of ATAC-Seq data, related to Figure 3. A. Fragment size distribution. B. Relative enrichment of genomic intervals. C. Correlation analysis. D. PCA analysis.


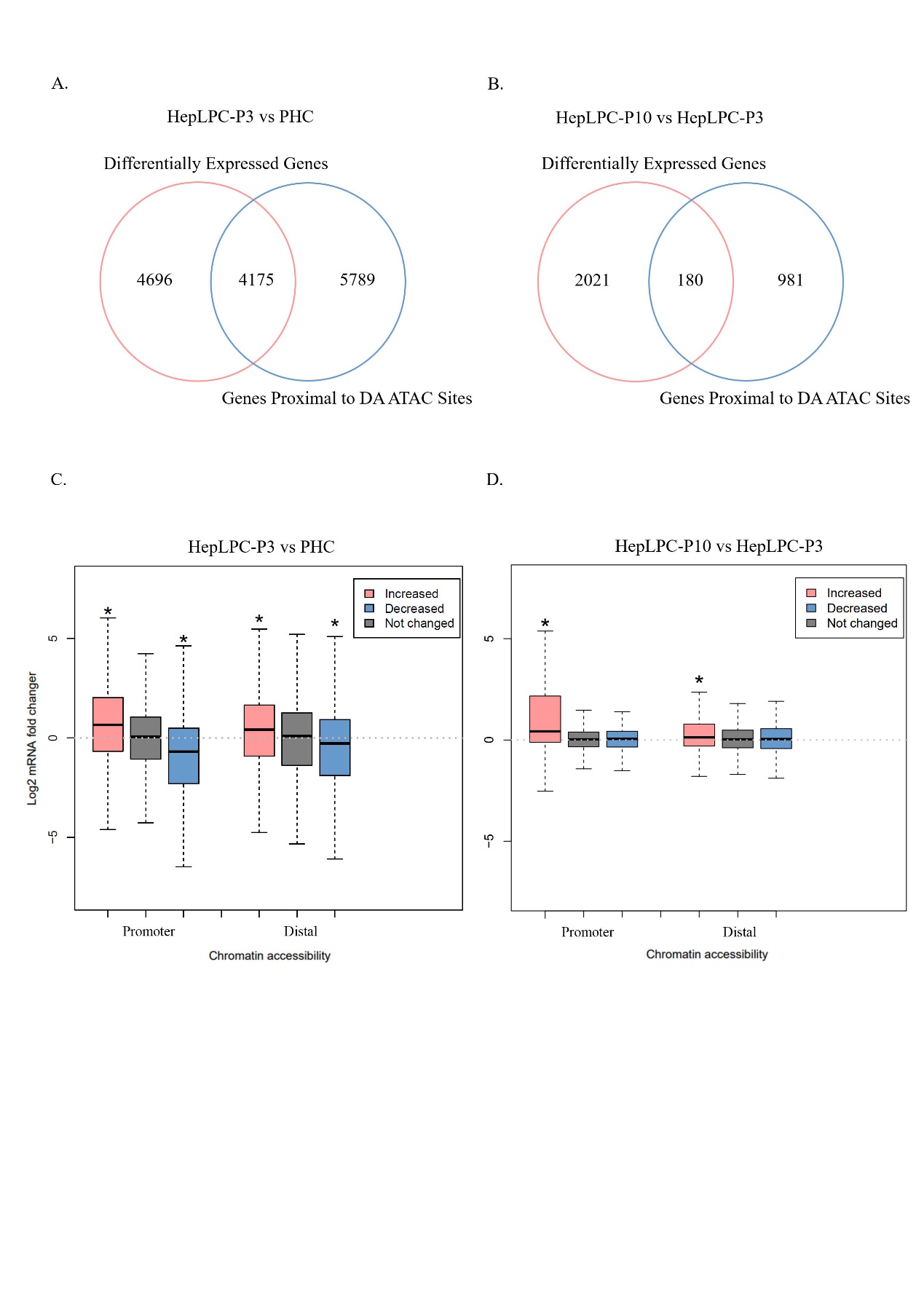


**Figure S3**. Association of expression levels and chromatin accessibility, related to Figure 3. A. Overlap of genes proximal to differentially accessible (DA) peaks and differentially expressed genes in HepLPC-P3 versus PHC (hypergeometric test p-value=8.80 x10^-23^). B. Overlap of genes proximal to differentially accessible (DA) peaks and differentially expressed genes in HepLPC-P10 versus HepLPC-P3(hypergeometric test p-value=2.04 x10^-11^). C-D. Association of changes in chromatin accessibility and gene expression in (C)HepLPC-P3 versus PHC and (D) HepLPC-P10 versus HepLPC-P3 showed that promoter and distal accessibility changes are associated with both gene activation and inhibition in HepLPC-P3 versus PHC (p<2.20x10^-16^), whereas only increased promoter (p=1.02 x10^-6^) and distal accessibility (p=8.81 x10^-5^) is correlated significantly with increased gene expression in HepLPC-P10 versus HepLPC-P3.

**
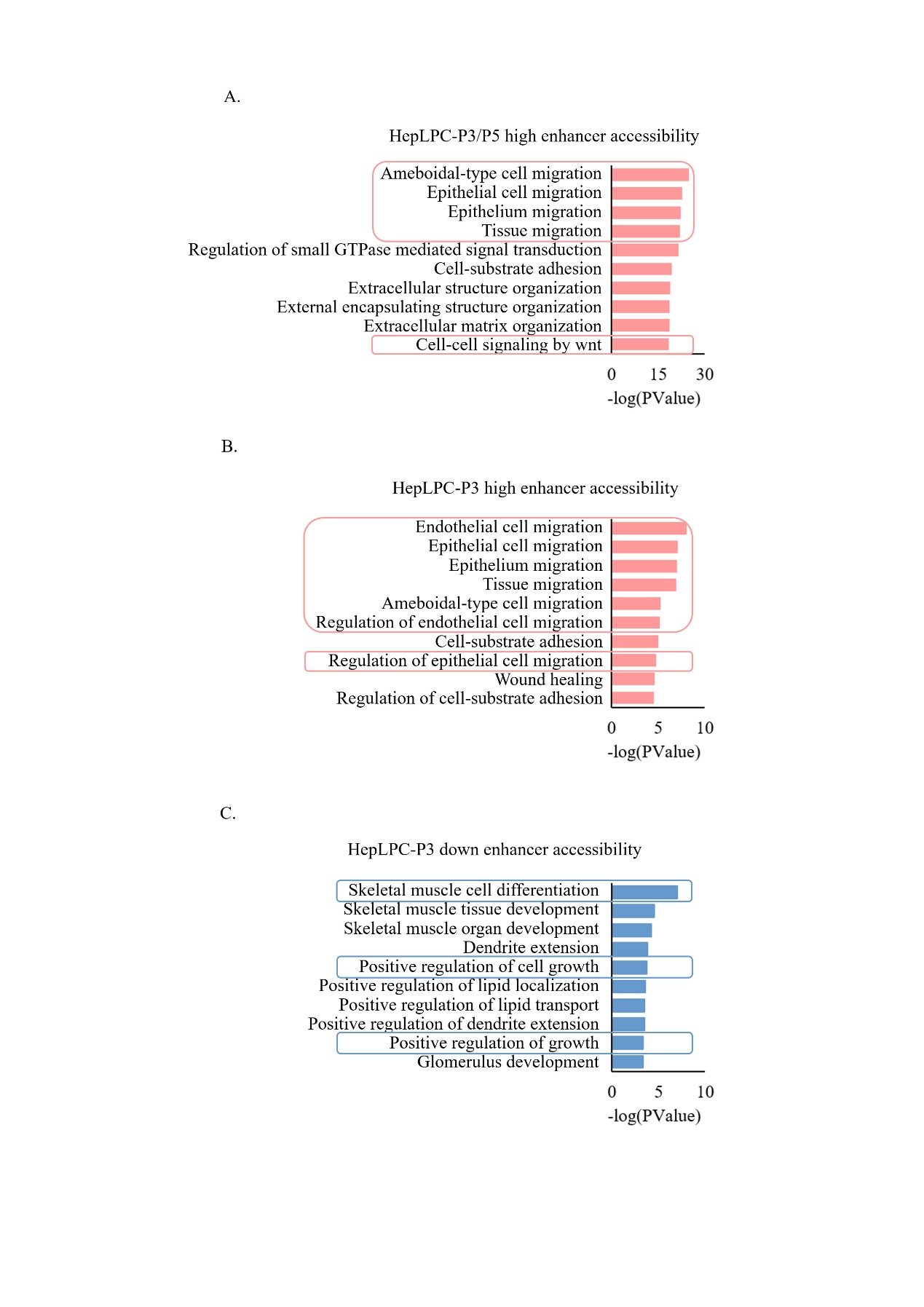
**

**Figure S4**. GO-BP analysis of genes with significantly differential distal accessibility among different cell states, related to Figure 4-5. A. GO-BP enrichment analysis of genes with increased distal accessibility in both HepLPC-P3 and HepLPC-P10 indicated that processes related to cell migration and cell-cell signaling by wnt were enriched. B. GO-BP enrichment analysis of genes with increased distal accessibility in HepLPC-P3 compared to PHCs and HepLPC-P10 indicated that processes related to cell migration were up-regulated. C. GO-BP enrichment analysis of genes with decreased distal accessibility in HepLPC-P3 compared to PHCs and HepLPC-P10 indicated that processes of cell differentiation and cell growth were down-regulated.

**
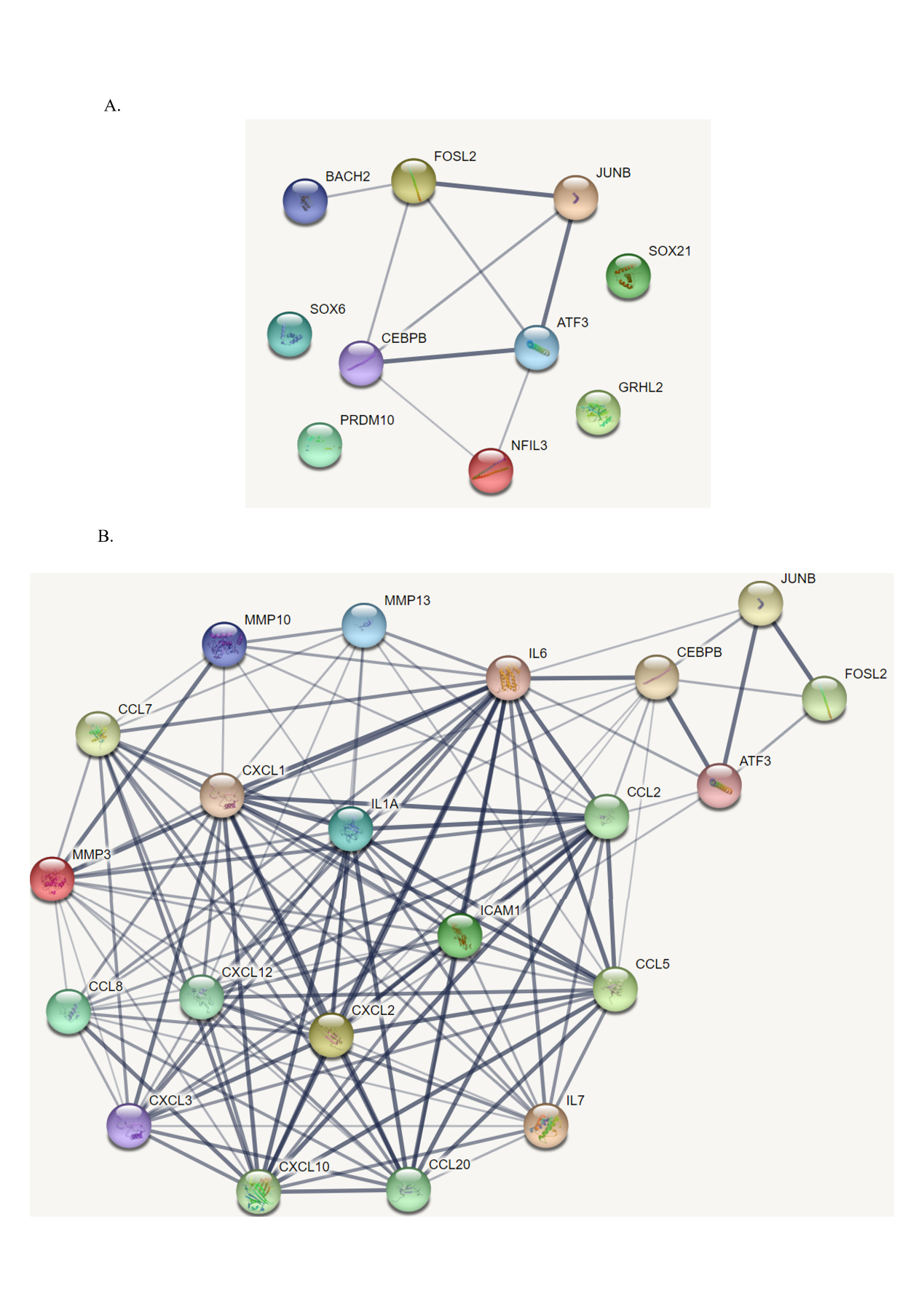
Figure S5** Protein-protein interaction networks of TFs. A. Interaction network of TFs in Figure 5D. B. Interaction network of core TFs (with high confidence 0.700) in A and SASP factors in Figure 2E. (The line thickness of the edge indicates the strength of data to support the interactions.)

**
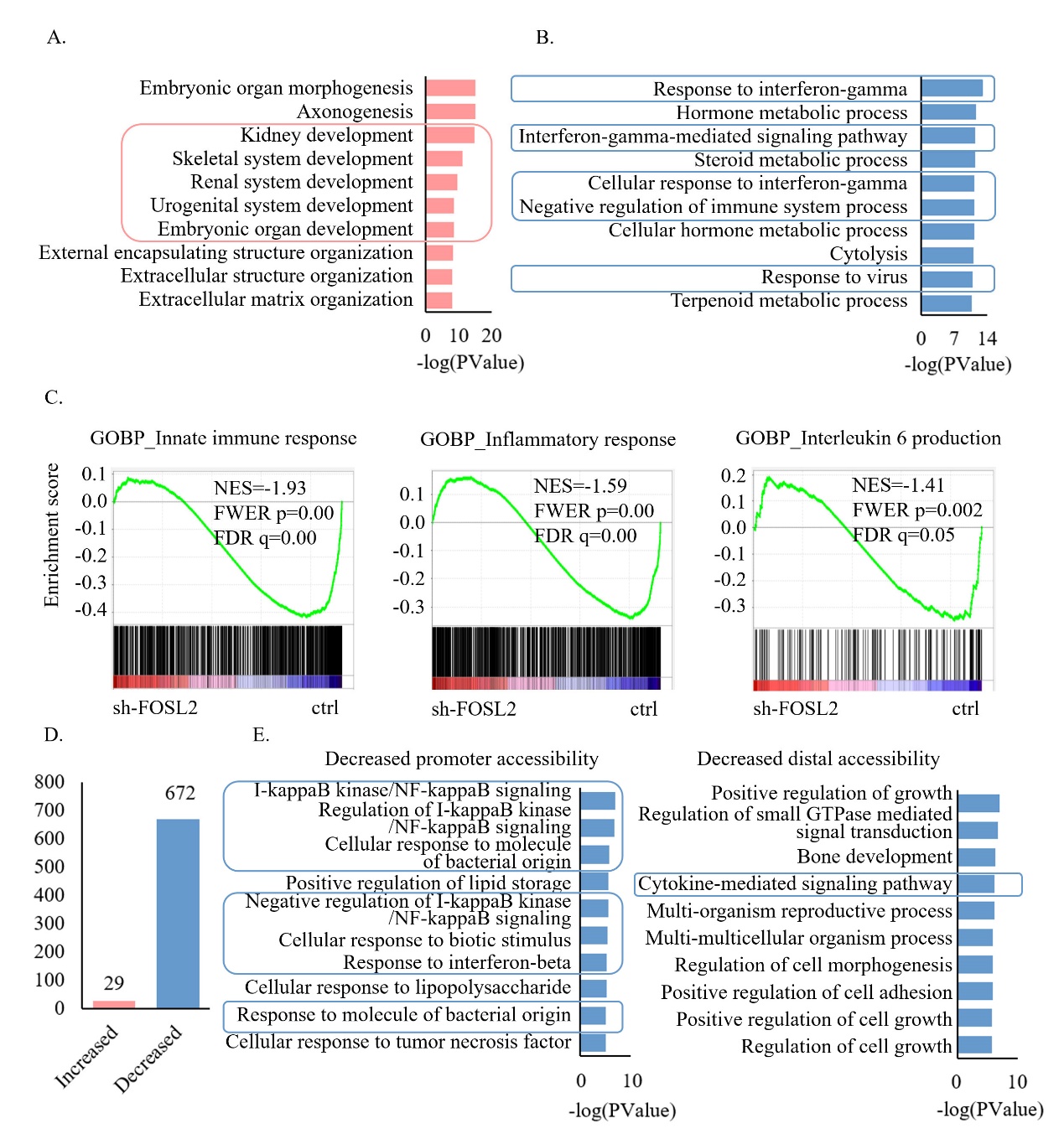
**

**Figure S6** Functional analysis of DEGs between sh-FOSL2 affected HepLPC-P10 and ctrl HepLPC-P10, related to Figure 6. A. GO-BP enrichment analysis of up-regulated genes in sh-FOSL2 affected HepLPC-P10 showed that developmental related pathways were enriched. B. GO-BP enrichment analysis of down-regulated genes in sh-FOSL2 affected HepLPC-P10 showed that inflammatory-related pathways were enriched. (red = up-regulated; blue = down-regulated). C. Gene set enrichment analysis (GSEA) showed that processes related to innate immune response, inflammatory response, and interleukin 6 production were repressed in sh-FOSL2 affected HepLPC-P10. D. Number of regions with significantly different accessibility between FOSL2 affected HepLPC-P10 and ctrl HepLPC-P10(FDR<=0.05). E. GO-BP enrichment analysis of genes with decreased promoter and distal accessibility in FOSL2 affected HepLPC-P10 compared to ctrl HepLPC-P10.

**Supplement Tables**

**Table S1** Primer sequences used for RT-qPCR

| Genes | Forward Primer(5’->3’) | Reverse Primer(5’->3’) |
| --- | --- | --- |
| EPCAM | AATCGTCAATGCCAGTGTACTT | TCTCATCGCAGTCAGGATCATAA |
| KRT19 | AACGGCGAGCTAGAGGTGA | GGATGGTCGTGTAGTAGTGGC |
| IL6 | ACTCACCTCTTCAGAACGAATTG | CCATCTTTGGAAGGTTCAGGTTG |
| SERPINE1 | ACCGCAACGTGGTTTTCTCA | TTGAATCCCATAGCTGCTTGAAT |
| P53 | CAGCACATGACGGAGGTTGT | TCATCCAAATACTCCACACGC |
| CXCL12 | ATTCTCAACACTCCAAACTGTGC | ACTTTAGCTTCGGGTCAATGC |
| CXCL3 | CGCCCAAACCGAAGTCATAG | GCTCCCCTTGTTCAGTATCTTTT |
| CCL2 | CAGCCAGATGCAATCAATGCC | TGGAATCCTGAACCCACTTCT |
| CEBPB | CTTCAGCCCGTACCTGGAG | GGAGAGGAAGTCGTGGTGC |
| ATF3 | CCTCTGCGCTGGAATCAGTC | TTCTTTCTCGTCGCCTCTTTTT |
| JUNB | ACGACTCATACACAGCTACGG | GCTCGGTTTCAGGAGTTTGTAGT |
| FOSL2 | CAGAAATTCCGGGTAGATATGCC | GGTATGGGTTGGACATGGAGG |
